# Febrile illness mapping—much of the world without data and without evidence-based treatments

**DOI:** 10.1186/s12916-020-01747-y

**Published:** 2020-09-21

**Authors:** Paul N. Newton, Philippe J. Guerin

**Affiliations:** 1 Infectious Diseases Data Observatory (IDDO), University of Oxford, Oxford, UK; 2 Lao-Oxford-Mahosot-Hospital-Wellcome Trust Research Unit, Microbiology Laboratory, Mahosot Hospital, Vientiane, Lao PDR; 3 Faculty of Infectious and Tropical Diseases, London School of Hygiene and Tropical Medicine, London, UK; 4 Boston University School of Public Health, Boston, USA; 5 Centre for Tropical Medicine and Global Health, Nuffield Department of Medicine, University of Oxford, Oxford, UK

## Background

The COVID-19 pandemic has highlighted the world-changing impact of infectious diseases to all 7.8 billion people. Our distant ancestors were aware of the enormous impact of epidemic fevers on their lives and the havoc that they wrought, as we continue to do today. However, before urbanisation, endemic infectious diseases would probably have had greater impact on people’s daily lives. Endemic infectious diseases, with the exception of those such as TB, HIV, malaria and dengue that have grabbed public, research and policy attention, have tended to be relatively neglected. Epidemics of Chikungunya, Ebola and Zika have hit the media spotlight in the last decade, but were often perceived as regional tropical issues. Endemic diseases may become epidemic and epidemic diseases endemic. We need to know what infectious diseases are where in order to inform prevention and public engagement programmes and treatment guidelines, both for specific pathogens and for syndromic empirical treatment, and to prioritise interventions and funding. Without such public health intelligence, we will wait for the next disaster blindfolded. The large scale implementation of affordable and accurate malaria rapid diagnostic tests (RDTs), usable by health workers with minimal training in rural malarious areas has demonstrated that malaria, although remaining a focally important cause of fever in many areas, has been overestimated as a cause. There have been decreases in malaria incidence in most endemic regions. Hence, there is a great need to understand the epidemiology of fevers other than malaria to inform many policy and implementation decisions.

The three papers (a fourth describing the data from China is awaited) in this series, that evolved from the work of Acestor et al. [1], describe systemic reviews of what we know about where different non-malarial fevers occur in south and southeast Asia, Africa and Latin America. A total of 69,104 records (29,558 from Asia, 16,523 from Africa, 23,023 from Latin America), published 1980–2015, were screened from 101 countries (19 in Asia, 48 in Africa, 34 in Latin America). Of these, 4099 (2410 in Asia, 1065 in Africa, 624 in Latin America) met selection criteria. A wide diversity of pathogens and their distribution are described (see [5] in press, [7] in press, [10] in press).

## The challenges of gaps and heterogeneity

The collection, categorisation and analysis of data in individual fever studies has followed very heterogeneous methodology and reporting, making these unprecedented reviews very challenging. We are in urgent need of objective consensus guidelines for the design and reporting of fever studies to aid the analysis of comparative epidemiology and thence to inform treatment policy. Adaptation of STROBE guidelines [17] to guide fever studies could help improve their rigour and impact. This issue has been highlighted for COVID-19 epidemiology research, and the great weight of our collective unpreparedness should be used to stimulate more thought and consensus building to allow better comparisons and translatable evidence. COVID-19 has inevitably stalled much non-COVID-related research—giving an opportunity to plan for post-pandemic fever research.

The term ‘undifferentiated’ fever is often used but without standardised definition. Its utility is in doubt as patients rarely have fever alone and some form of differentiation is common; for example, scrub typhus rarely presents without headache and myalgia. How undifferentiated is ‘undifferentiated’ [13, 16]? Few of the studies used the same inclusion criteria, with great variation in aspects such as age ranges and the upper limit of the age of children. The frequent lack of control volunteers makes it especially hard to understand the clinical importance, or otherwise, of key potential pathogens such as respiratory viruses.

The maps describe the current situation and highlight key areas for where more data, standardised reporting and sharing of information are needed. Many studies of the causes of fever reported in recent years are from large urban conurbations, rather than the vast tracts of the rural tropics that remain febrile *terra incognita.* There are large gaps in the evidence base as illustrated by these reviews, in areas with significant numbers of vulnerable people, such as central Africa and the highlands of the Southeast Asian Massif. The conventional wisdoms, for example that the *Japanese encephalitis virus* and *Orientia tsutsugamushi*, the agent of scrub typhus, are purely diseases of the Asia-Pacific, have been dramatically overturned in the last decade [11, 14]. Hence, aside from the large geographical gaps, pathogen description globally needs to be improved and techniques such as niche mapping used to predict where pathogens may be, to guide investigation as to their true global distribution [8]. Such modelling would be greatly enhanced by representative standardised data collection.

## Linkage between surveillance and research

The data behind the reviews and map represent academic studies and how they relate to national surveillance programmes, where they exist, is difficult, and often impossible to assess. There are few regularly updated international repositories of diverse infectious disease epidemiological data; only for a few pathogens with vertical programmes, such as malaria, TB, HIV, influenza and some neglected tropical diseases. The dysfunctional flows for the reporting of confirmed COVID-19 patients and variable denominators illustrate the unpreparedness of many countries or regions. The amalgamation of national surveillance data, academic papers and systems such as ProMed, in openly accessible databases and national mapping systems would allow a clearer understanding of what is where and amongst who, to inform prevention, detection and treatment. If these academic data reflect surveillance data, an unknown but very substantial proportion of global populations remain ‘invisible’ as they live beyond surveillance systems due to insufficient infrastructure. Linkage of academic and surveillance data seems a lost opportunity. Investment in innovative strategies facilitating infectious disease surveillance in remote rural communities, through primary care provision of RDTs, could become an important component of targeted prevention and treatment and poverty reduction. However, there remain relatively few accurate and affordable RDTs for our great diversity of fevers. Investment in highly sensitive, specific, accessible and affordable diagnostic tests to allow fever diagnosis amongst communities currently without access, in both rural and remoter and urban areas, would help fill these gaps and facilitate development of algorithms for empirical therapy of fevers in primary care. Yet, it is unlikely that such RDTs could cover the whole range of infectious diseases, and using the reviews published here could help the prioritisation of both their development and the deployment on a regional basis, guided by the epidemiology of pathogens circulating in those areas.

There are no up-to-date inventories of what pathogens are present in our world or per country. One of the last attempts at a global synthesis was the three volumes of Simmons et al. [12], but the Americas were not apparently included, presumably because they escaped continental land warfare in World War II. The GIDEON system collates global data but is a commercial system [4], unaffordable by most in LMICs. Armed forces include medical intelligence organisations, and WHO is leading the Epidemic Intelligence from Open Sources (EIOS) system. Has not the time come for an accessible global system linking nations for sharing, curating and mapping infectious disease data, surveillance, academic and lay, both endemic and epidemic, to inform national, regional and global policy and implementation? National registers and data on when, where and how pathogens are diagnosed in countries can be used for discovery curve analysis to predict how many pathogens may be found within a country based on past accrual ([2] in press).

## Informing algorithms

A key aim of fever studies should be to inform national and subregional treatment guidelines and algorithms, empirical and pathogen specific, but apart from work in Tanzania, there has been little such overt translation [3, 9, 16]. How algorithms do and should vary across geography has been little investigated. Through modelling spatial differences in treatment algorithms across the long north-south range of Laos, White et al. [15] suggested that only moderate spatial variation would result in spatially explicit sub-national fever treatment algorithms being significantly more efficacious than national algorithms.

Large fever studies are expensive in terms of staff time and the expense of mass laboratory testing [6]. Few reference laboratories can handle the splurge in samples in a timely manner at an affordable cost and there are few such reference laboratories in endemic countries. These difficulties lead to the need to compromise between the ideal testing strategy for the estimated patient sample size and what is financially possible. Again, our collective unpreparedness for COVID-19 must lead to change to improve this, led by countries that have successfully managed a rapid roll out of testing.

## Conclusion

The COVID-19 pandemic is demonstrating the vital importance of infectious diseases to policy makers and the populations they represent and gives us the opportunity and the obligation to re-think how we do things and learn from collective failure. The SARS-Cov-2 virus is almost certainly here to stay as an endemic pathogen and will need to be integrated into prevention and treatment algorithms. These mapped data suggest that we should plan now for consensus guidelines on the design and reporting of fever studies, improving data sharing and identifying areas of the world with sparse epidemiological information, for the population size, to prioritise for investigation, and diverging from the ‘usual places’, and optimising use of these data to inform empirical treatment algorithms.

## Data Availability

Not applicable
